# Topo IV is required to allow replisomes to converge and complete replication on the chromosome

**DOI:** 10.1371/journal.pgen.1011857

**Published:** 2025-09-08

**Authors:** Parisa D. Mokhtari, Tannos Seyedjavadi, Thulni A. Liyanaarachchi, Charmain T. Courcelle, Justin Courcelle

**Affiliations:** Dept of Biology, Portland State University, Portland, Oregon, United States of America; The University of North Carolina at Chapel Hill, UNITED STATES OF AMERICA

## Abstract

The ability to complete DNA replication as replisomes converge has recently been shown to be a highly regulated, multi-enzymatic process. Converging forks also are likely to generate unique supercoiled, tangled, or knotted substrates. These structures are typically resolved by one of the four topoisomerases encoded by *Escherichia coli*. However, identifying the cellular substrates and specific function for these essential enzymes which contain overlapping biochemical activities has remained challenging. Here, we show that Topo I and Topo IV are required to allow converging forks to complete chromosome replication. Impaired Topo I function leads to amplifications where forks converge, whereas inactivation of Topo IV prevents forks from converging and produces a dramatic loss of this chromosome region. The results are consistent with previous studies suggesting Topo I suppresses illegitimate initiations in the terminus region by disrupting R- and D-loops and demonstrate a specific requirement for Topo IV acting before replication completes to allow convergent forks to reach their doubling point. We propose that the positive supercoils arising between convergent forks are converted to precatenanes and resolved by Topo IV, when diminishing space may preclude gyrase from binding and functioning.

## Author summary

DNA topoisomerases resolve twists, knots, and other topological problems arising from the need to unwind the double-helical DNA molecule in order to replicate or transcribe the genome. *E. coli*, like most organisms, including humans, encode several topoisomerase enzymes. Many of these are essential to maintain viability and are useful targets in several chemotherapeutic and antimicrobial treatments. However, identifying the specific role of each enzyme in the cell can prove challenging since their fundamental nature can lead to multiple phenotypes and they often have partially overlapping biochemical activities. Here we show that both Topo I and Topo IV of *E. coli* play a role in the critical process of completing DNA replication on the chromosome when two replication forks converge. The function of Topo IV appears specific, acting prior to the completion of replication, and is required to allow the converging forks to reach their doubling point.

## Introduction

Cells tightly regulate the processes of DNA replication initiation, elongation and completion to ensure that each cell receives an identical copy of the genetic information. Whereas initiation and elongation have been well characterized (reviewed in [1,2]), less is known about how cells complete replication. To complete replication accurately, cells must encode an enzymatic system that is capable of recognizing pairs and joining the nascent strands of converging replication forks faithfully, without gain or loss of genetic information. The failure to complete even a single replication event would be expected to result in a loss of genomic stability, or cell lethality. Yet, this reaction must be remarkably efficient, occurring thousands of times per generation along the chromosomes of human cells. Given this critical role, it is not surprising that this final step is tightly regulated and controlled by a complex multi-enzymatic pathway [3–7].

*E. coli* represents a useful model to dissect how completion occurs as its genome contains a single 400- kb region where replication forks converge opposite to its bidirectional origin of replication (reviewed in [8]). Completion events are contained within this region by Tus, which binds to termination (*ter*) sequences and prevents replication forks from progressing beyond this region [9,10]. Although Tus confines completion to this region, it does not appear to directly participate in the reaction since mutants lacking Tus or *ter* sites complete replication normally [11,12].

Whereas Tus does not appear to directly participate, several gene products have been identified that impair the completion reaction when mutated and result in abnormalities specifically at loci where replication forks converge [3,5,13,14]. Current models suggest that the converging replisomes transiently bypass each other, creating a partially over-replicated region that contains a third copy of the genetic information. These over-replicated intermediates can be observed in the absence of exonucleases, which allow these intermediates to persist, both in vitro and in vivo [3,5,7,15,16]. RecG is a helicase with branch migration activity that limits the amount of over-replication and appears to prevent these DNA end intermediates from re-initiating replication [3,17–19]. It is thought that the over-replicated intermediate is first incised by SbcCD and ExoI, a cohesin-like structure-specific nuclease and 3’ to 5’ exonuclease [5,7,20], before the heterotrimeric RecBCD helicase-nuclease complex further processes and resects this region [5,6,20]. RecBCD function is also thought to be needed before DNA Ligase and Polymerase I can join the nascent strand ends of convergent forks, as in its absence, extensive degradation in this chromosome region occurs, severely compromising viability [5,13]. Importantly, and perhaps surprisingly, the completion reaction does not require recombination, as *recA* mutants complete replication normally [5]. However, RecA is required for many of the aberrant amplifications and duplications that arise when completion is impaired, suggesting that recombination occurring under these conditions is directly associated with genomic instability [5–7,20].

Completion events would also be expected to generate a number of topological challenges on the DNA. During normal replication, negative and positive supercoils accumulate behind and in front of the progressing fork DNA, respectively [21,22] and reviewed in [23]. Additionally, if the fork DNA is free to swivel, positive supercoils can be relieved, generating precatenanes in the newly replicated sister chromatids behind the fork as these become twisted around each other [24–26]. As replisomes converge, the space available for enzymes to access and resolve these twists and tangles diminishes. Resolution of such structures may require unique enzymes to access these regions or structures. Additionally, over-replicated intermediates at loci where the forks converge would potentially compound these issues should swiveling of the DNA ends be prevented by tethering to proteins or membranes. Cells typically process these topological constraints using DNA topoisomerases, making it likely that some of these enzymes will play a role in the completion of replication. However, at present, it remains unclear, how topological complexities generated during completion are processed or resolved.

*E. coli* encodes two type I and two type II DNA topoisomerases. These enzymes form transient protein-DNA complexes that break either one (type I) or both (type II) strands, before swiveling and rejoining the DNA to relieve or introduce twists in the double-stranded helix (reviewed in [23]).

Topo I and III, encoded by *topA* and *topB* respectively, are type I topoisomerases [27,28]. Topo I is an essential enzyme that relaxes negatively supercoiled plasmids *in vitro* and *in vivo* [29–34]. It is thought to act during both transcription and replication, by reducing negative supercoiling behind the replisome and transcription machinery [35]. Suppressor mutations that restore viability appear to either alleviate transcriptionally generated R-loops, rpoB*35, *rnh*, or decrease negative supercoiling during replication via reduced gyrase activity or upregulated Topo III or Topo IV activity [14,34,36–41]. Consistent with a general role in these essential processes, hypomorphic or suppressed *topA* mutants have altered transcription profiles and reduced replication rates [38,39,42,43].

Topo III is the only nonessential topoisomerase in *E. coli* and mutants exhibit normal growth [28]. In vitro, its activity is stimulated by RecQ and relaxes negative supercoils and resolves precatenane plasmid substrates [14,44–46].

Gyrase, encoded by *gyrA* and *gyrB*, and Topo IV, encoded by *parE* and *parC*, are Type II topoisomerases ([36,47,48] and reviewed in [23]). In vitro, gyrase catalyzes negative supercoils in an ATP-dependent manner and is thought to operate ahead of the replication fork [22,49,50]. The counteracting activities of gyrase and Topo I are thought to balance and maintain supercoiling levels in the overall genome. Inactivation of DNA gyrase slows the rate of DNA replication, consistent with the idea that the protein travels with the replication machinery to relieve the positive supercoils generated ahead of the replication fork [51–54].

Topo IV has been proposed to have many roles that partially overlap with gyrase and the enzymes share ~40% homology [36]. However, Topo IV is significantly more efficient in decatenating plasmid molecules than DNA gyrase and this appears to be related to its function in vivo [21,22,49,55]. Temperature-sensitive Topo IV mutants accumulate catenated plasmids following Topo IV inactivation [21,56]. Some evidence based on single-molecule, fluorescence microscopy has suggested Topo IV travels behind the replisome during replication, although a genomic analysis of Topo IV cleavage sites indicate that its activity increases in the terminus region on the chromosome [57–59].

Here, we focused on which of these topoisomerases participate in completing DNA replication on the chromosome. Characterizing how topoisomerases participate in specific cellular events can be challenging because they are often essential, and can affect a range of metabolic activities that elicit pleiotropic phenotypes in vivo. Plasmid minichromosomes, which contain a bi-directional origin of replication, have been used effectively to characterize aspects of how converging replication forks complete replication in vivo [9,15,20,60–62]. We characterized topoisomerase mutants for their ability to propagate these substrates and correlated it with events observed on the *E. coli* chromosome. We show that both Topo I and Topo IV are required to maintain substrates containing convergent forks and that Topo IV plays a specific and critical role in allowing replisomes to reach their meeting point on the chromosome.

## Results

### Topo I, but not Topo III, enhances the ability of replication forks to converge on plasmids

To begin to assess the role of the type I DNA topoisomerases in completing DNA replication, we compared the ability of *topA2103* and Δ*topB* mutants to maintain minichromosome plasmid, pCL01, which contains a bi-directional origin and replicates using two replisomes, to that of plasmid pBR322, which replicates using a single replisome from a uni-directional origin and therefore does not require that replisomes converge [63,64]. The ability to maintain the two-replisome plasmid has been shown previously to correlate with the cell’s ability to maintain the chromosome region where replication forks converge [20,61]. We reasoned that if a topoisomerase contributes to the ability of replication forks to converge, then propagation of the 2-replisome plasmid would be more severely impaired than that of the 1-replisome plasmid.

**Fig 1:**
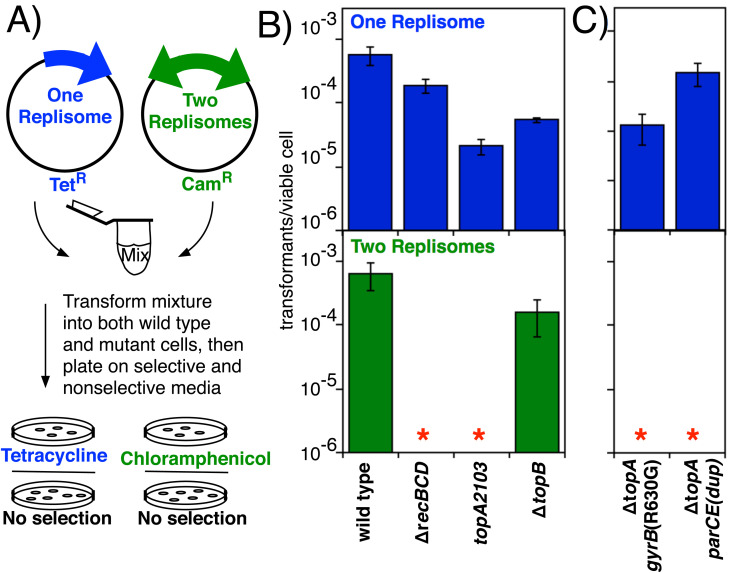
Mutations in *Topo I but not Topo III impair the ability to maintain plasmids containing convergent replication forks.* A) Illustration of how transformation frequencies were measured. A mixture containing the 1- and 2-replisome plasmids was transformed into both parental and mutant strains that were then plated on selective and nonselective media. The ratio of cells containing each plasmid was then determined by counting colonies following overnight incubation. B) Loss of Topo I activity impairs the ability to maintain plasmids containing convergent replication forks. The number of transformants per viable cell for the 1-replisome (pBR322) and 2-replisome (pCL01) plasmid is plotted for wild-type, ∆*recBCD, topA2103,* and ∆*topB*. C) Other Topo I mutants similarly compromise the ability to maintain plasmids containing convergent replication forks. The number of transformants per viable cell for the 1-replisome (pBR322) and 2-replisome (pCL01) plasmid is plotted for ∆*topA gyrA*(R630G) and ∆*topA parCE*^*dup*^*.* ∆*topA parCE*^*dup*^ contains a chromosome duplication encompassing the *parC parE* region*.* Plots represent the average of between three and nine independent experiments. Error bars represent the standard error of the mean. Asterisks indicate transformants were below our limit of detection.

To determine transformation efficiency, a single solution containing both plasmids was transformed into all strains (Fig 1A). Transformation of both the 1-replisome and 2-replisome plasmid was similar in the parental strain (Fig 1B). By contrast, when the plasmid mixture was transformed into Δ*recBCD* mutants, the transformation frequency of the 2-replisome plasmid was severely reduced. As noted above, RecBCD is required to maintain the chromosomal region where replication forks converge, demonstrating that the 2-replisome plasmid can be used to identify gene products participating in the completion reaction [5,20]. Most reports suggest Topo I is essential [34,38,41,65]. Therefore, we initially examined a *topA2103* mutant along with a Δ*topB* mutant. *topA2103* was isolated as a spontaneous frameshift mutation that removes the protein’s C-terminal domain, but retains partial function. As shown in Fig 1B, transformation of the 2-replisome plasmid was significantly reduced in *topA2103* but not Δ*topB* mutant strains, although the 1-replisome plasmid could still propagate in both strains.

Since the *topA2103* mutant retains partial function, we could not eliminate the possibility its presence has a dominant effect that poisoned or impaired some aspect of the completion reaction. Therefore, despite reports that *topA* is essential, we attempted to construct deletion mutants of Topo I in our parental background, eventually obtaining two separate isolates. However, upon sequencing, both Δ*topA* mutants were found to have secondary, presumable suppressor, mutations in other topoisomerases. Isolate 1 contained an arginine to glycine mutation at amino acid 630 in *gyrB*, whereas isolate 2 contained an ~ 900 kb genomic duplication encompassing *parE* and *parC* region. However, since the *topA* deletion was present and the strains remained viable, we continued characterization of these strains. As shown in Fig 1C, transformation of the 2-replisome plasmid was impaired in both Δ*topA* strains, even in the presence of these suppressor mutations. Taken together, the observations argue against *topA2103* having a dominant effect and suggest that Topo I, but not Topo III, activity promotes the propagation of substrates containing two replisomes.

### Topo IV, but not DNA gyrase, enhances the ability of replication forks to converge on plasmids

**Fig 2:**
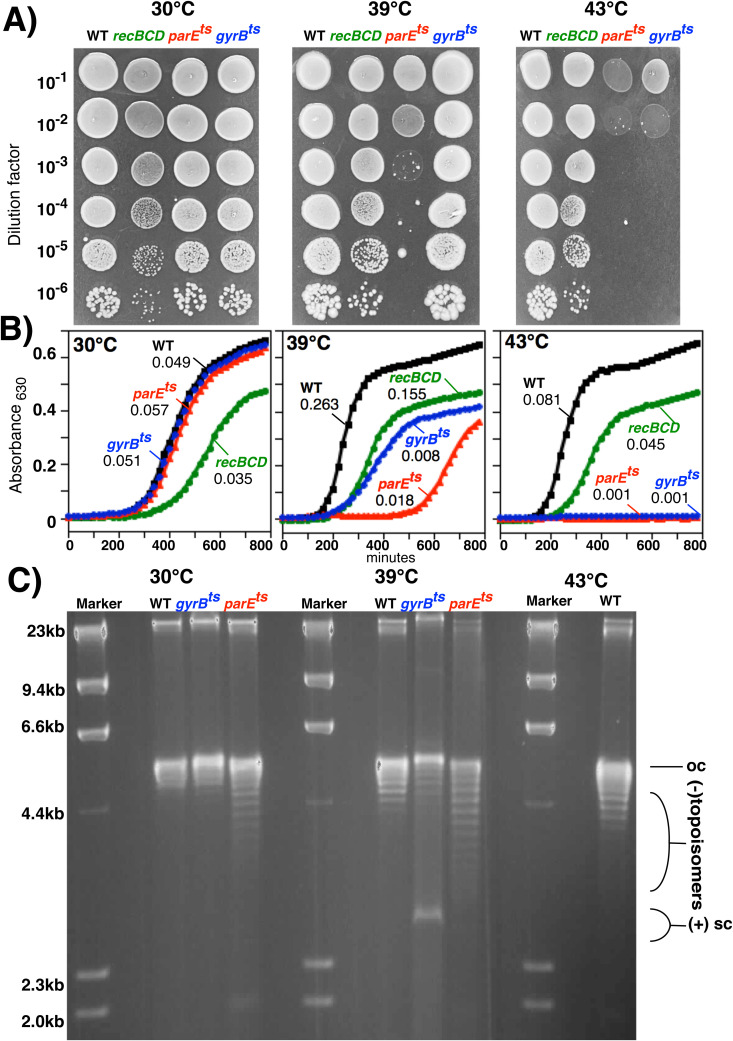
Properties of *gyrB*^ts^ and *parE*^ts^ strains at the permissive, semi-permissive, and restrictive temperatures used in this study. A) Viability of *gyrB*^ts^ and *parE*^ts^ strains at 30ºC, 39ºC, and 43ºC. Ten-µl drops of 10-fold serial dilutions were spotted onto nonselective plates for each strain and grown at the indicated temperature. B) Growth of *gyrB*^ts^ and *parE*^ts^ strains at 30ºC, 39ºC, and 43ºC. The Absorbance at 630 nm is plotted for each strain at the indicated temperature over time. C) Strains containing plasmid pBR322 were grown at the indicated temperature before plasmids were purified and examined following electrophoresis in a 1.1% agarose gel containing 8 µg/ml choroquine. Oc- open circle, sc – supercoils, M- lambda Hind III molecular size markers.

Both type II DNA topoisomerases are essential for viability. However, temperature-sensitive *gyrB* and *parE* mutants exist which are viable at 30°C but lose viability at 42°C [36,66]. We first confirmed that these mutant alleles remained temperature sensitive when present in our parental background. Both mutants retained viability and grew similar to wild-type cells at 30°C (Fig 2). However at 39°C, *parE*^ts^ mutants began to lose viability, grew at a slower rate, and Topo IV function became impaired as evidenced by elevated levels of negative topoisomers on plasmids purified from these cells. Similarly, *gyrB*^ts^ mutants also grew more slowly at 39°C and gyase function was impaired as evidenced by the appearance of positively supercoiled plasmids purified from these cultures. In contrast to *parE*^ts^, *gyrB*^ts^ mutants retained viability at this temperature. At 43°C, both mutants lost viability and failed to grow. *gyrB*^ts^ mutants remained viable up to 42°C, and we were unable to identify a temperature where these mutants lost partial viability as seen with *parE*^ts^. For the purposes of comparison and as a control, *recBCD* mutants were also examined and shown not to be temperature sensitive, but did grow more slowly, an observation which has been previously reported and shown to relate to the impaired ability to complete replication [5,20]. Thus, although both enzymes are partially inactivated at the semipermissive temperature, they produce phenotypically distinct outcomes for the cell. However, we cannot rule out that *gyrB*^ts^ and *parE*^ts^ viability differences reflect different degrees of protein inactivation. Since viability was lost at 43°C, we elected to use 39°C as a semi-permissive temperature to examine if these enzymes compromise the ability to maintain substrates containing convergent forks when their activity is partially impaired.

**Fig 3:**
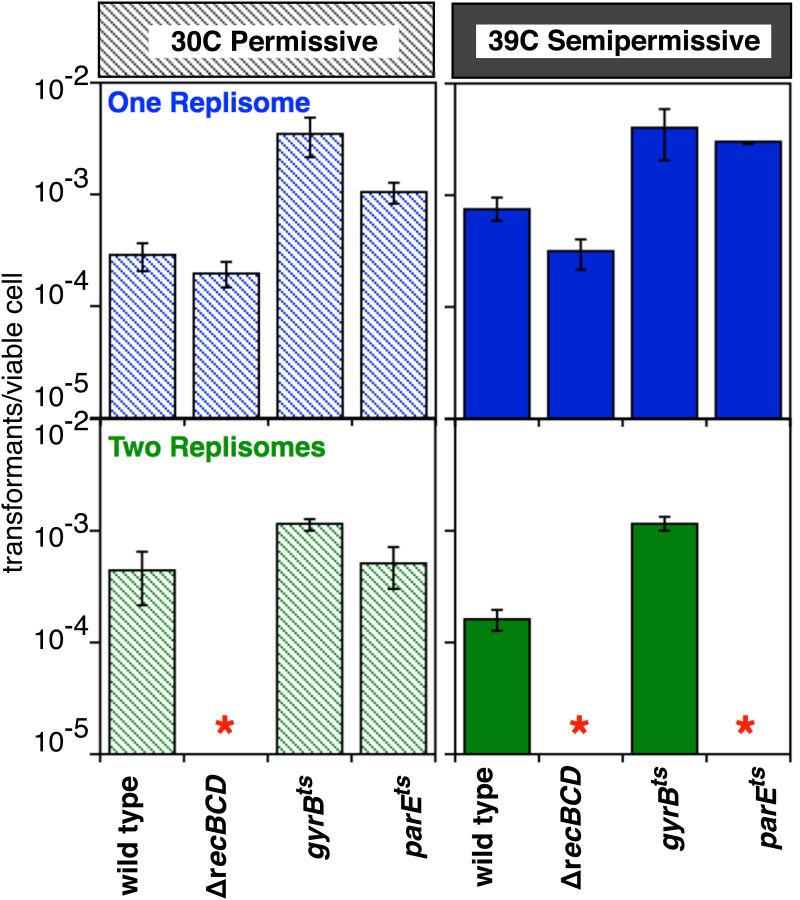
Loss of Topo IV activity impairs the ability to maintain plasmids containing converging replication forks. The number of transformants per viable cell for the 1-replisome and 2-replisome plasmids is plotted at the permissive (30°C) and semi-permissive (39°C) temperatures for wild-type, ∆*recBCD*, *gyrB*^ts^ and *parE*^ts^ mutants. Transformants per viable cell were determined as in Fig 1. Plots represent the average of three independent experiments. Error bars represent the standard error of the mean. Asterisks indicate transformants were below our limit of detection.

Following transformation of the plasmid mixture into these strains at the 30°C permissive temperature, the parental, *gyrB*^ts^ and *parE*^ts^ strains were all able to propagate both the 1- and 2-replisome plasmids. Similarly, Δ*recBCD* mutants remained impaired for the 2-replisome plasmid as before (Fig 3). However, differences emerged at the 39°C semi-permissive temperature. *gyrB*^ts^ remained capable of propagating the 1- and 2-replisome plasmids; while transformation of the 2-replisome plasmid into *parE*^ts^ was compromised relative to the 1-replisome plasmid (Fig 3), indicating that impaired Topo IV function affects the cell’s ability to maintain substrates containing convergent replisomes.

### Impaired Topo IV activity generates abnormal replication intermediates when replisomes converge

**Fig 4:**
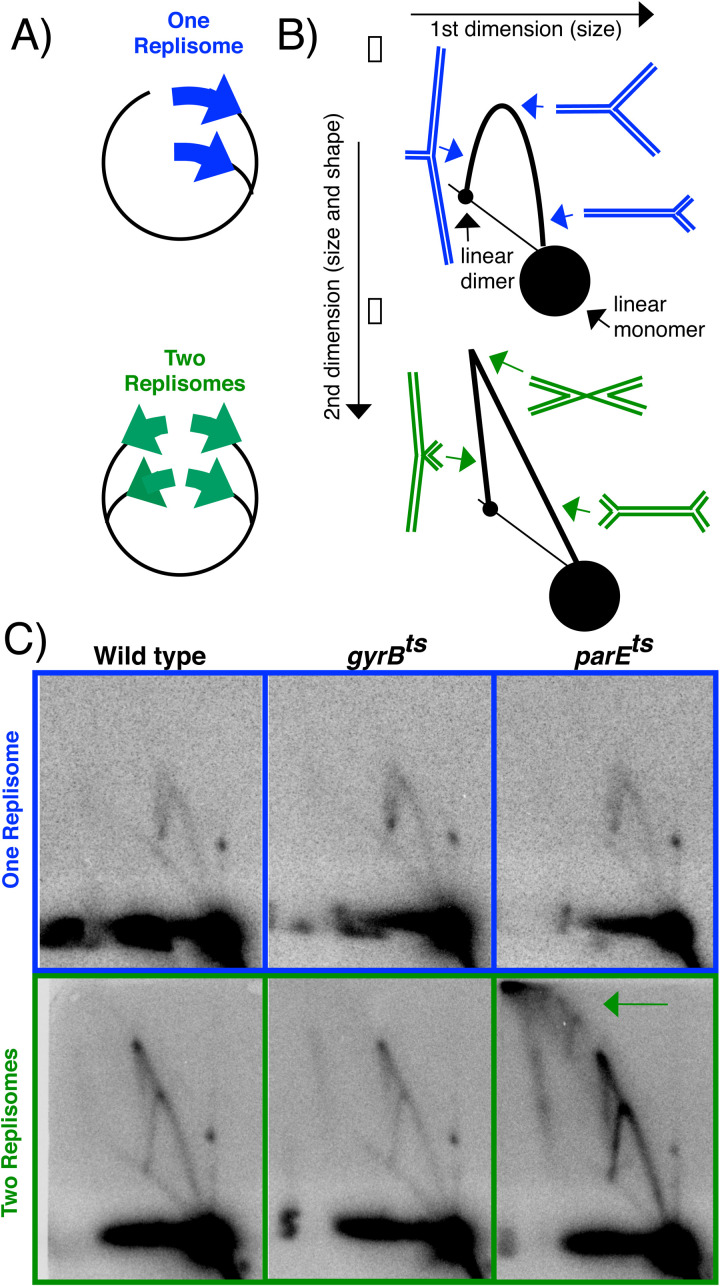
Topo IV mutants accumulate abnormal replication intermediates on substrates with convergent replication forks. A & B) Illustration depicting the expected migration pattern of 1- and 2-replisome plasmids when linearized at the origin of replication. Following restriction enzyme digestion, nonreplicating plasmids run as a linear monomer fragment, forming the prominent spot observed in gels. Plasmids with unidirectional origins have replicating fragments that form Y-shaped molecules, which form an arch that extends out from the linear monomer fragment and returns to the linear dimer fragment. Plasmids with bidirectional origins have replicating fragments that form double Y- or X-shaped molecules. Double Y-shaped molecules form a line that extends out from the linear monomer fragment, while X-shaped molecules appear as a line extending up from the linear dimer fragment to a point where the two lines meet. C) When Topo IV activity is compromised, large abnormal replication intermediates accumulate on substrates containing convergent replication forks. DNA was purified from wild-type, *gyrB*^ts^ and *parE*^ts^ cells containing the 1- or 2-replisome plasmids and analyzed by 2D-agarose gel electrophoresis. (Top panels) Normal replication intermediates are observed in wild-type, *gyrB*^ts^ and *parE*^ts^ cells containing plasmids with one replisome. (Bottom panels) Normal replication intermediates are observed in wild-type and *gyrB*^ts^ cells containing plasmids with two replisomes. However, abnormal intermediates accumulate in *parE*^ts^ cells that correspond to large branched fragments containing single-stranded regions (green arrow). Gels shown represent one of three independent experiments for each strain.

The results above demonstrate that impaired Topo I or Topo IV function compromises the cell’s ability to maintain plasmids containing convergent replisomes. Although we were unable to identify conditions where *topA* mutants could propagate the 2-replisome plasmid, the ability to transform *gyrB*^ts^ and *parE*^ts^ mutants with the 2-replisome plasmid at the permissive temperature provided an opportunity to examine the replication intermediates associated with this mutation. To this end, we used 2-dimensional (2D) agarose gel electrophoresis, a technique that can be used to visualize the structure of replicating DNA fragments in vivo [67,68]. Total genomic DNA was purified from growing cultures that contained either the 1-replisome or 2-replisome plasmid. The DNA was then digested with a restriction enzyme that cleaved the plasmids at their origin of replication, and the plasmid DNA was analyzed by Southern analysis following 2D agarose gel electrophoresis. In this technique, nonreplicating plasmids migrate as a linear fragment, forming the prominent spot observed on the 2D gel. Replicating fragments migrate more slowly because of their larger size and nonlinear shape. On plasmids containing a unidirectional origin, these replicating fragments form a simple Y-arc that extends out from the linear fragment. Plasmids with a bidirectional origin of replication produce fragments with double Y-shapes, migrating in an inverted V-shape that extends up from the linear monomer fragment and down to the linear dimer fragment (Fig 4A & 4B). In theory, the two-replisome plasmid should contain only double-Y structures. In practice however, when the two replisomes initiate at slightly different times, molecules will initially appear as a single-Y structure, rather than a double-Y, which can be seen in the Southern analysis of the molecules. To demonstrate that both replisomes are active on the plasmid, two *ter* sequences, which block replication progression in an orientation-specific manner [9], were cloned into plasmid pCL01 and then re-analyzed by 2D agarose gel analysis. S1 Fig shows that replication arrest events are observed at both *ter* sequences, demonstrating that both replisomes are active on the 2-replsome plasmid.

In the case of the 1-replisome plasmid, both *gyrB*^ts^ and *parE*^ts^ strains formed normal Y-shaped intermediates, similar to the parental strain. Similarly, the replication intermediates of 2-replisome plasmid formed the expected inverted-V shaped in the parental and *gyrB*^ts^ strains. However, in *parE*^ts^ mutants, replication of the 2-replisome plasmid led to the formation of large, multimeric intermediates containing either one or two branch points (Figs 4C and S2). The abnormal intermediates likely comprise molecules having single-strand regions or gaps, since these multimers remain resistant to digestion by the restriction endonuclease. The observation that abnormal intermediates only accumulated on plasmids with two replisomes, but not one replisome, argues that suboptimal Topo IV activity impairs the ability to complete replication specifically on substrates containing convergent replication forks.

### Loss *of* Topo IV activity impairs *the* ability *of* convergent replisomes *to* complete chromosome replication

**Fig 5:**
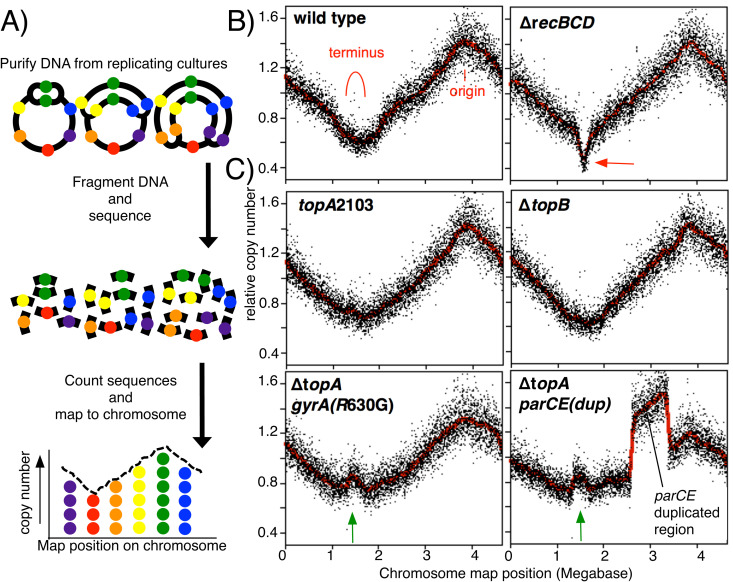
Impaired Topo I function leads to amplifications at loci where replication forks converge on the chromosome. A) An illustration depicting how replication profiles are determined. Genomic DNA is purified from replicating cultures, fragmented, and sequenced using high throughput sequencing. The relative number of sequence reads within each kilobase of the genome is then plotted. B) The profiles of wild-type cells and *recBCD* mutants are plotted. In replicating cultures of wild-type cells, replication proceeds bidirectionally from the origin and completes in the terminus region, as indicated. In the absence of *recBCD*, the nascent DNA ends fail to join and remain susceptible to exonucleolytic degradation, leading to a loss of sequence reads in this region of the genome (red arrow). C) *topA* mutants, but not *topB* mutants, exhibit amplification in the terminus region. Profiles of *topA*2103, ∆*topB,* ∆*topA gyrA*(R630G) and ∆*topA parCE*^*dup*^ are plotted. ∆*topB* does not lead to alterations within the terminus region of the chromosome. Amplified terminus region with increased copy number in ∆*topA gyrA*(R630G) and ∆*topA parCE*^*dup*^ is observed (green arrow). Profiles shown represent one of two independent experiments for each strain.

**Fig 6:**
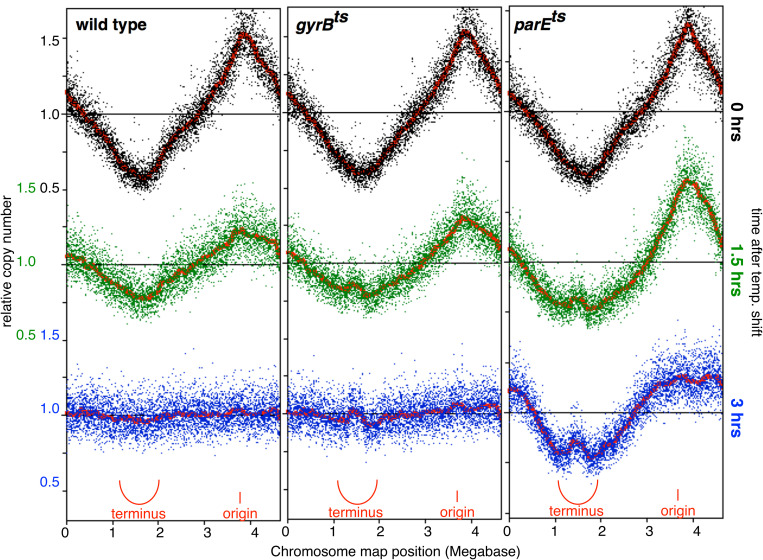
Inactivation of Topo IV leads to a specific inability to replicate the terminus region of the chromosome. Replication profiles of growing wild-type, *gyrB*^ts^, and *parE*^ts^ cells are plotted at 0, 1.5, and 3 hrs after temperature shift from 30°C to 43°C. In actively growing wild-type cultures, the copy number of sequences decreases inversely with distance from the origin until reaching the terminus region. After three additional hours, replication in the culture has largely ceased, and the copy number of all sequences across the chromosome is equal. Following inactivation of gyrase (in *gyrB*^ts^ mutants) the ongoing rounds of replication are able to complete, similar to wild-type cells. However, inactivation of Topo IV (in *parE*^ts^ mutants) prevents the completion of ongoing replication events, leading to a loss of sequence reads in the terminus region and an inability to maintain the region of the chromosome where forks converge. Profiles shown represent one of two independent experiments for each strain.

The results above demonstrate that Topo I and Topo IV activity are required to maintain plasmid substrates that contain convergent replication forks. To determine if these enzymes function similarly on the chromosome, we profiled replication across the genome in growing parental and mutant cultures. To profile replication, genomic DNA was extracted from growing cultures, fragmented, and then sequenced using high-throughput sequencing. The frequency each sequence occurs is then plotted relative to its position on the chromosome (Fig 5A). In parental cultures, the frequency of sequences surrounding the origin is highest because it replicates first. The frequency of sequences then decreases inversely with their distance from the origin, until reaching the region where forks converge and replication completes (Fig 5B). Consistent with previous work, in Δ*recBCD* mutants which fail to join the strands of convergent forks, a dramatic loss of sequences is observed specifically at the locus where replication completes (Fig 5B and [6,20]).

Using this technique, we first examined the profiles of the type I topoisomerase mutants. In *topA2103* or Δ*topB* mutants, the profile of replication looked similar to that of the parental strain. However, in the Δ*topA* strains which contained suppressors in DNA gyrase or Topo IV, a modest increase in copy number, or over-replication, of the region where replication forks converge was observed (Fig 5C). The results are consistent with those obtained with the 2-replisome plasmid and suggest that the presence of Topo I but not Topo III is required to allow replication to complete normally.

To examine the role of essential type II topoisomerases, temperature-sensitive *gyrB* and *parE* mutants were grown at 30°C then shifted to 43°C for 90 or 180 minutes to follow the progression of the ongoing replication in the cultures. At these time points, the genomic DNA was then purified and sequenced as described above. Under these conditions (i.e., three hours of replication at 43°C), the cultures were reaching maximum density and cell growth and DNA replication had ceased. In the replication profiles of wild-type cultures, this is observed as a damping in the difference between the frequency of origin and terminus sequences, as new initiations become less frequent by 90 minutes. By 180 minutes, new initiations have ceased and ongoing rounds of replication have completed, leaving equal frequencies at all regions around the chromosome (Fig 6). Following a shift to the semi-permissive temperature, the profile of the *gyrB*^ts^ mutants resembles and resolves in a manner similar to wild-type cultures with the exception of a modest over-replicated region that persists at the 180 minutes time point. Interestingly, in the *parE* ^ts^ replication profile, 90 minutes after shifting to the nonpermissive temperature, a similar modest over-replicated region is present at the loci where replication completes. Additionally however, the regions in the latter half of the chromosome are under-represented, suggesting that replication is not progressing through this portion of the chromosome. By 180 minutes when replication has ceased, this unreplicated region becomes clear and pronounced, leaving the terminus region unreplicated. The results demonstrate a specific defect in completing replication when Topo IV activity is inactivated. Taken together, the plasmid and chromosomal observations imply that the activities of Topo I and Topo IV promote the efficient completion of replication when replication forks converge. When these activities are absent or impaired, genetic instabilities arise specifically at these loci.

## Discussion

The results demonstrate that impaired Topo I or Topo IV function can lead to an inability to maintain substrates containing convergent replication forks and that this correlates with abnormalities on the chromosome at sites where replication forks converge. In the case of Topo I, extensive work from the Drolet lab and others suggests the effect is largely related to the stabilization of R-loops, three-stranded nucleic acid structures, consisting of an RNA-DNA hybrid and a displaced single-stranded DNA [14,41,51,69–77]. These three-stranded structures form when transcribed RNA remains hybridized with the DNA template, and are stabilized under conditions when negative supercoiling increases. D-loops, three-stranded DNA structures, are similarly stabilized under these conditions. The hybridized strands of R-loops and D-loops can serve as primers that lead to RecA-dependent illegitimate initiations of replication [14,41,75,78,79]. Early studies often characterized R- and D-loop replication events as novel, or cryptic origins of replication based on copy number analysis of these regions in replicating cultures, and found they either localized to the origin or the terminus regions, where replication forks meet [80–85]. Loss of Topo I function was found to lead to an increase in these initiation events and could bypass the requirement for DnaA, encoding the essential replication initiation factor at *oriC* [14,41,73–75,78,84,86]. Consistent with these studies, we observe an over-replication of the terminus in suppressed strains lacking Topo I activity (Fig 5).

Although the over-replication of the terminus region in *topA* mutants is likely associated with elevated chromosomal instabilities, it seems unlikely to account for the essential role of Topo I in viability. While mutants which fail to maintain (under-replicate) the terminus region often compromise cell growth and viability, *E. coli* are generally tolerant of mutations that lead to over-replication in the terminus region [3,5,7,87]. Mutants lacking exonucleases, or the RecG helicase severely over-replicate the terminus region, yet remain viable and grow well. Thus, while the specific nature of these over-replicated intermediates remains unknown, it is clear that their presence does not necessarily compromise cell growth or viability. One possible reason for the essentiality of Topo I that has been proposed is that the supercoiling alters and detrimentally affects cellular transcription in an essential way. This could occur directly by altering promoter recognition and efficiency, or impairing elongation [35,42,88]. It could also be an indirect effect of the stabilized R-loops, which may block essential processes of transcription or even replication progression, or a combination of both [14,35,41,54,69,89]. Excessive R-loops are associated with cellular toxicity. Growth defects in *topA* mutants can be partially suppressed by RNase HI overexpression [39,73]. However, another study, which relied on a plasmid maintenance assay, did not observe this RNase HI suppression, although *rnhA* mutants did exacerbate the *topA* effect [65]. Our observation that *topA* mutants can have issues maintaining plasmids in some contexts (Fig 1), could suggest why this latter study did not observe such an effect. The pleiotropic effects Topo I has on both transcription and replication make deciphering the essential function targeted by this enzyme difficult to identify and *topA* lethality may result from a combination of these possibilities. Nevertheless, we observed that Topo I activity was required to maintain plasmids containing convergent replication forks. Considering that it was not required on substrates containing a single replisome, our results suggest some aspect of Topo I activity is contributing to the success or efficiency of events occurring during the completion of replication. Of interest is the modest over-replicated intermediate or transient amplifications observed between promixal *ter* sites in *topA* deletion mutants that contain suppressor mutations in gyrase or Topo IV. These amplifications are also observed in the *gyrB*^ts^ and *parE*^ts^ mutants alone but is reduced in the nonsuppressed, *topA*2103 point mutation, raising the possibility that the amplification relates to alterations in type II activities of gyrase or Topo IV, rather than Topo I. Several other genes associated with the normal completion of replication exhibit similar over-replicated intermediates when mutated, although the structural form of these intermediates remains unclear [3,7,13,16,20,87]. Curiously, while the over-replication is likely associated with increased genomic instability and mutation, it does not affect viability or growth. Additionally, these mutants, including *recG* and *sbcCDxonA*, remain capable of replicating plasmids containing convergent replication forks [7,20]. In contrast, mutants which under-replicate, or fail to maintain this region, such as *recBCD* or *sbcCDxonA* lacking RecA, compromises growth and viability, similar to that seen in *parE*^ts^ mutants. Notably, these mutants also fail to propagate plasmid substrates containing convergent replication forks.

**Fig 7:**
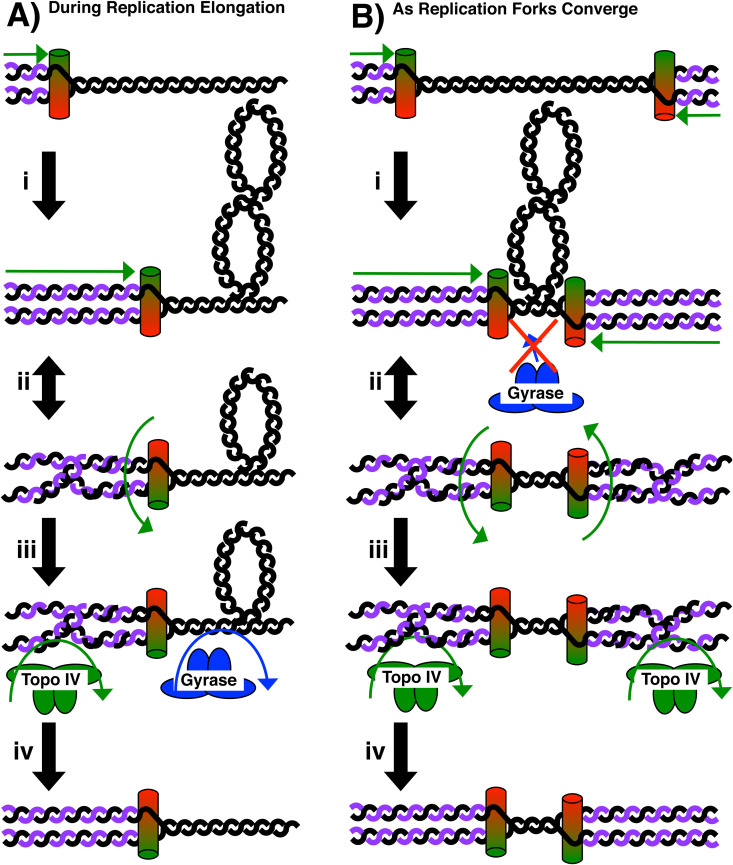
Model for the role of Topo IV in completing replication on the chromosome. A) During the normal progression of replication (i), unwinding of the DNA helix generates positive supercoiling, or overtwisting ahead of the DNA fork (ii). Rotating of the replisome and fork DNA can relieve the positive supercoiling, but generates twists, or precatenanes behind the fork (iii). Most data suggest that gyrase relieves positive supercoiling ahead of the fork, while Topo IV relieves precatenanes behind the fork (iv). B) When replisomes converge (i), gyrase is unable to relieve positive supercoiling between completing replisomes due to limited binding space (ii) This makes relieving this tension entirely dependent on the resolution of the precatenanes behind the fork (iii) by Topo IV (iv).

An essential function for Topo IV in allowing replication to reach the site where completion occurs was also observed. Topo IV inactivation leads to a specific loss of plasmids containing convergent replication forks which correlates with an accumulation of large unresolved plasmid intermediates containing single-strand regions (Figs 3–4). On the chromosome, inactivation of Topo IV manifests as an inability to replicate the terminus region of the chromosome, a phenotype which is likely to account for its essential role in viability (Fig 6). A number of genetic and biochemical studies from the Cozzarelli lab distinguished the substrate preferences for gyrase and Topo IV resolving positive supercoils and catenanes, respectively [21,47,49,56]. In some elegant biochemical studies using *oriC*-plasmid substrates, Hiasa and Marians took this further and showed that Topo IV was preferentially acting on the precatenanes behind the fork [22]. Interestingly and consistent with the observations on the chromosome reported here, they show that although either gyrase or Topo IV is sufficient to allow replication fork progression (Fig 7A), precatenane removal by Topo IV was essential to support the final stages of DNA replication. Notably, the under-replicated region spans several megabases, encompassing almost half of the chromosome, which may suggest that precatenane accumulation in the absence of Topo IV may reach a critical point which eventually inhibits replication elongation more generally.

Several studies have since supported the conclusion that Topo IV acts behind the fork throughout the replication cycle. Using fluorescently tagged version of a replisome protein (SSB), a replisome-trailing protein (SeqA), and Topo IV to localize these proteins in living cells, the Skarstad lab inferred that Topo IV followed the replication machinery during the cell cycle [59]. High throughput sequencing studies have also mapped Topo IV binding and cleavage sites across the genome, while the Sherratt lab found that Topo IV expression levels correlated with the time sister chromatids remained associated [57,58,90]. A mutation in *dnaX* that prevents Topo IV association with the travelling replisome was also shown to result in filamentation and segregation defects [91].

Whereas, the above studies indicate Topo IV travels throughout the genome during replication, other observations suggest it has a unique activity at the end of replication. The genomic mapping of Topo IV binding and cleavage sites shows an enrichment of its activity in the terminus region proximal to the *dif* site [57–59]. Consistent with this, Topo IV interacts directly with FtsK and cleaves proximal to the *dif* site in a XerCD resolvase-dependent manner [92,93]. The activities promoted by *dif* and FtsK, chromosome dimer resolution and cell septation, respectively, are events that occur after chromosome replication has completed. This would seem to indicate that either the Topo IV interactions are involved in signaling or regulating these later events, or that Topo IV also has resolving functions which operate after replication has completed [94]. Irrespectively, the lack of replication of the terminus region on the chromosome following Topo IV inactivation establishes an essential role for the enzyme in allowing convergent replication forks to progress to their meeting point. During the normal progression of DNA replication, gyrase is thought to act ahead of the fork to relieve the positive supercoils. However, the ability of Topo IV to resolve precatenanes can also relieve this tension if the replication forks swivel, which converts positive supercoiling ahead of the fork to precatenanes located behind the fork (Fig 7A). As demonstrated by Hiasa and Marians, either activity is sufficient to allow replication fork progression [22]. However, as the replication forks converge, the space remaining ahead of the fork diminishes, and at some point is likely to preclude gyrase from binding and acting. At this point, we would speculate that further progression of the converging replisomes may then depend entirely on the activity of Topo IV relieving this tension through the precatenane resolution which occurs behind the convergent replication forks (Fig 7B). In the absence of Topo IV, this superhelical tension would remain, preventing the replication forks from converging and replication of this region on the chromosome.

Several studies have also suggested that Topo III may act at convergent replication forks. In an elegant biochemical study by Suski and Marians, Topo III, encoded by *topB*, was shown to be capable of resolving convergent replication forks on *oriC* plasmids in the presence of RecQ [95]. Additionally, Topo III localizes at the replication fork and is active on precatenanes, similar to Topo IV [95]. It also acts as a multicopy suppressor of Topo IV mutants [62]. However, similar to others, we were unable to discern a phenotype in *topB* mutants associated with completing DNA replication, either on plasmids containing convergent forks, or on the chromosome in vivo [14]. While the observations indicate the enzyme is not required for this reaction to occur, its association with the RecQ family of helicases, and the replication fork suggests an important cellular function that our current assays or approaches have been unable to reveal.

Whereas completing replication occurs only once on the bacterial chromosome, it occurs hundreds to thousands of times per generation along the multi-origin, linear chromosomes of eukartyotic organisms as has been previously characterized [96–101]. Many of these bacterial proteins have structural or functional homologs in eukaryotes, suggesting that this fundamental aspect of cellular metabolism will be conserved throughout evolutionarily divergent organisms.

## Materials and methods

### Strains and plasmids

The parental strain used for this study is BW25113, which has the genotype *rrnB3* ∆*lacZ*4787 *hsdR*514 ∆(*araBAD*)567 ∆(*rhaBAD*)568 *rph*-1. Strains used in this study are listed in Table 1. BW25113 *ymjA*::FRT-minikan (JW1288), BW25113 *yieL*::FRT-minikan (JW5612), and BW25113 *topB*::FRT-mini-kan (JW1752) are part of the Keio single gene knockout mutant collection [102]. CL4079 (BW25113 *sbcD*::FRT-mini-kan *topA2103*) containing a single base pair deletion in *topA* at nucleotide 2103 was isolated as a spontaneous secondary mutation that resulted in plasmid instability in laboratory stocks of BW25113 *sbcD*::FRT-mini-kan. To construct a BW25113 *topA2103* mutant, the kanamycin-resistance cassette from the *sbcD* deletion mutant was first removed using FLP recombinase expression from the pCP20 plasmid as described previously [103,104]. *topA2103* was then linked to a kanamycin-resistance cassette approximately 25 kb away by P1 transduction of *ymjA*::Kan from JW1288 and selecting for resistance to kanamycin and plasmid instability to generate CL5110 (BW25113 *sbcD*::FRT *topA2103 ymjA*::FRT-mini-kan). CL5111 was made by P1 transduction of *topA2103 ymjA*::FRT-mini-kan from CL5110 into BW25113. A second *topA* mutation, ∆(*topA*-*cysB*), was similarly linked to a kanamycin-resistance cassette by P1 transduction of *ymjA*::kan from JW1288 into RFM475 (31) and selecting for kanamycin resistance and cysteine auxotrophy to generate CL5427. CL5379 (large colony morphology) and CL5380 (small colony morphology) were made by P1 transduction of ∆(*topA*-*cysB*) *ymjA*::kan from CL5427 into BW25113. All strains were further verified by genome sequencing. CL5428 was made by P1 transduction of *parE*ts::Tn10 from MG1655 *parE*ts::Tc [105] into BW25113 then selected for loss of tetracycline resistance using chlortetracycline hydrochloride as previously described [106] to generate CL5429. A temperature-sensitive mutation in *gyrB* was linked to a kanamycin-resistance cassette by P1 transduction of *yieL*::kan, located approximately 25 kb away from *gyrB*, from JW5612 into RFM445 [39] and selected for kanamycin resistance and temperature sensitivity to generate CL5328. CL5431 was made by P1 transduction of *gyrB*ts *yieL*::kan from CL5328 into BW25113. CL5429 (BW25113 *parE*ts) and CL5431 (BW25113 *gyrB*ts *yieL*::kan) were verified for temperature sensitivity.

**Table 1:** Bacterial strains used in this study.

Bacterial Strain	Genotype	Source
BW25113 Parent	(*araD*-*araB*)567, Δ*lacZ4787*(::*rrnB*-3), λ-, *rph*-1, Δ(*rhaD*-*rhaB*)568, *hsdR*514	[104]
CL4079	BW25113 *sbcD*::FRT-mini- kan *topA2103*	This study
CL5110	BW25113 *sbcD*::FRT *topA2103 ymjA*::FRT-mini- kan	This study
CL5111	BW25113 *topA2103 ymjA*::FRTminikan	This study
CL5250	BW25113 *recBCD*::kan	[20]
CL5328	N99 *gyrB*ts *yieL*::kan	This study
CL5379	BW25113 ∆(*topA*-*cysB*) *ymjA*::kan *gyrA*(R630G)	This study
CL5380	BW25113 ∆(*topA*-*cysB*) *ymjA*::kan *parEC*(duplication)	This study
CL5427	N99 ∆(*topA*-*cysB*) *ymjA*::kan	This study
CL5428	BW25113 *parE*^ts^::Tn10	This study
CL5429	BW25113 *parE*^ts^ *tet*^s^	This study
CL5431	BW25113 *gyrB*^ts^ *yieL*::FRT- minikan	This study
JW1752	BW25113 *topB*::FRTminikan	[102]
JW1288	BW25113 *ymjA*::FRTminikan	[102]
JW5612	BW25113 *yieL*::FRTminikan	[102]
RFM445	*rpsL galK2 gyrB221*(cou^R^) *gyrB203*(Ts) ∆*trpE* ∆*lac74*	[39]
RFM475	*rpsL galK2 gyrB221*(cou^R^) *gyrB203*(Ts) ∆*trpE* ∆(*topA*- *cysB*)*204 trp*^* + *^∆*lac74*	[39]
*parE*(Ts)	MG1655 *parEts*::Tc	[105]

Plasmid pBR322 used in this study contains a unidirectional origin of replication, which has been described previously [63] and confers ampicillin and tetracycline resistance. pCL01 plasmid used in this study contains a bidirectional origin of replication from bacteriophage λ [64] and ampicillin- and chloramphenicol-resistance cassettes [20]. Plasmids used in this study are listed in Table 2.

**Table 2:** Plasmids used in this study.

Plasmids	Genotype	Source
pBR322	*amp*^R^ *tet*^R^ ColE1 origin	[63]
pCL01	*amp*^R^ *cam*^R^ λ origin	[20]

### Plasmid assay

Electro-competent cells were prepared by growing a 100-fold dilution of a fresh overnight culture in 50-mL Luria-Bertani broth without salt (LB, no NaCl) to an OD_600_ of 0.4. Strains were grown at 37°C or at 30°C for the temperature-sensitive strains. Cells were pelleted, washed with 50-mL of ice-cold 10% glycerol then resuspended in 200–300 μL of ice-cold 10% glycerol, and immediately frozen at -80°C. pBR322 and pCL01 were combined in a single mixture such that equivalent transformants per viable cells were obtained for each plasmid in wild-type cultures. The same plasmid mixture was then used for all strains analyzed. 50-μL of competent cells were mixed with the plasmid mixture, electroporated at 2.5 kV, capacitance 25 μF, resistance 200 Ohms, and allowed to recover in 1-mL SOC media for 60 minutes at 37°C. Transformation reactions were then 10-fold serially diluted and spotted in triplicate 10-μL aliquots on sets of LB plates with no antibiotic, 15 μg/mL tetracycline, or 20 μg/mL chloramphenicol supplementation to determine the number of viable cells, pBR322, and pCL01 transformants, respectively. Plates were incubated overnight at 37°C and colonies were counted the next day. The relative transformation efficiency was calculated as the ratio of transformants per viable cells in the mutants to the transformants per viable cells in wild-type cultures.

For temperature-sensitive strains, transformation efficiencies were compared between the permissive (30°C) and semi-permissive (39°C) temperatures. 50-μL of competent cells were mixed with plasmid mixture and electroporated using the conditions described above, except 2-mL of SOC was added to the cells post-electroporation, then each transformation reaction was divided equally into two 1-mL aliquots that were incubated for 1 hour at 30°C or 39°C. Following the 1-hour recovery period, cells were serially diluted and plated as described above. Plates were incubated overnight at each respective temperature and the next day, viability and transformation efficiency was determined as described above.

### DNA purification for 2D gel electrophoresis

Overnight cultures of cells containing pBR322 or pCL01 grown in LB supplemented with 15 μg/mL tetracycline or 20 μg/mL chloramphenicol, respectively, were diluted 100-fold into 10-mL of LB and grown to an OD_600_ of 0.5 at 37°C or 30°C for temperature-sensitive strains. A 0.75-mL aliquot of cells was then collected into an equal volume of ice-cold NET buffer (100 mM NaCl; 10 mM Tris, pH 8; 10 mM EDTA, pH 8), pelleted then flash frozen, and stored at -80°C.

Cell pellets were resuspended in 120-μL of lysis buffer (1 mg/mL lysozyme and 0.2 mg/mL RNaseA in 10 mM Tris, pH 8.0; 1 mM EDTA, pH 8.0) and incubated at 37°C for 30 minutes. Then, 10-μL of 10 mg/mL Proteinase K and 10-μL of 20% Sarkosyl were added to each sample, and incubation was continued at 37°C for 1 hour. Samples were extracted once using four volumes of phenol-chloroform, followed by two volumes of chloroform, then dialyzed on a 47-mm Millipore 0.05-μm pore disk floating on 200-mL of 1 mM Tris, pH 8; 1 mM EDTA, pH 8.0 for 30 minutes.

### 2D gel electrophoresis and Southern analysis

Purified DNA from cells containing the pBR322 plasmid were digested with PvuII restriction enzyme, while purified DNA from cells containing the pCL01 plasmid were digested with BstEII restriction enzyme. Following a 24-hour incubation at 37°C, digested DNA was extracted with one volume of chloroform then loaded onto a 0.4% agarose gel in 1x TBE and electrophoresed at 1 V/cm for 14–16 hours. For the second dimension, lanes were excised, rotated 90°, recast in a 1% agarose gel in 1x TBE, and electrophoresed at 6.5 V/cm for 6–8 hours.

DNA from the gels was transferred to Hybond N+ nylon membrane. Plasmid DNA was detected using either ^32^P-labeled pBR322 or pCL01 plasmid DNA prepared by random-primer labeling (Agilent Technologies) using alpha-^32^P-labeled-dCTP (3000 Ci/mmol) (Perkin-Elmer), and visualized using a STORM PhosphorImager (Molecular Dynamics) and analyzed using ImageJ software.

### Sample preparation for replication profiles

Overnight cultures of each strain were diluted 100-fold into 10-mL of LB and grown to an OD_600_ of 0.5 at 37°C or 30°C for temperature-sensitive strains. Then, 0.75-mL aliquots of each culture were collected in an equal volume of ice-cold NET buffer (100 mM NaCl; 10 mM Tris, pH 8; 10 mM EDTA, pH 8). Cells were pelleted, flash frozen, and stored at -80°C.

For replication profiles following temperature shift in wild-type and temperature-sensitive strains, overnight cultures were diluted as described above and grown to an OD_600_ of 0.3 at the permissive temperature of 30°C. Half of the cells were then collected by filtration on 0.45-μm membranes (Fisher Scientific) and resuspended in media pre-warmed to the non-permissive (43°C) temperature, while the remaining culture was allowed to continue growing at 30°C. 0.75-mL aliquots of each culture were collected in an equal volume of ice-cold NET buffer immediately following resuspension in pre-warmed media and after a further 90- and 180-minutes incubation at 30°C, or 43°C, as described above. Stationary-phase wild-type cultures were grown in LB for 36 hours at 37°C for use as a normalization control.

### Replication profiles

Genomic DNA was prepared as described for 2D gel electrophoresis above. Genomic DNA samples were sequenced using paired-end, 51-bp, bar-coded reads prepared and run using seqWell library prep kits (seqWell, Beverly MA, USA) and Illumina Next Seq 2000 (Illumina, San Diego, CA, USA). Breseq software is a command line tool that identifies mutations in DNA, relative to a reference genome [107]. This software was used to identify mutations between each strain and the wild-type BW25113 reference genome. Sequencing reads were aligned to the reference genome using Bowtie 1.0.0 software. Aligned reads were characterized to determine the nucleotide frequency at each position. Sequences were divided into 1-kb bins and plotted using a custom Python script developed by our lab. Copy numbers for each strain were normalized against the wild-type stationary culture. Plots represent the relative copy numbers at each genomic location in 1-kb bins and depict the replication profile for each strain.

## Supporting information

S1 Fig Both replisomes are active on the two-replisome plasmid. ter sequences were cloned into the plasmid, pCL01 in a manner that mimics their position on the chromosome, and the plasmids were analyzed by 2D agaose gel analysis as before. Two prominent sites where replisomes arrest, corresponding with the location of the ter sequences, are observed on the plasmid containing ter sequences (right panel) but not in their absence (left panel). Arrest sites are indicated by arrows. DNA preparation and 2D agarose gel analysis was performed as in Fig 4.
(EPS)

S2 Fig High-order intermediates accumulate on 2-replisome plasmids in *parE*^ts^ mutants. The percent of each replication intermediate in wild type, *gyrB*^ts^, and *parE*^ts^ mutants is shown. The color-coded areas quantified from Fig 4 are indicated. Nonreplicating plasmids, red; Y-structure and Early double-Y- structures, green; Late double-Y structures, blue; Complex, high order intermediates, purple.
(EPS)

S1 Data Data files for reproducing graphs contained in the figures. (XLSX)
